# Limitations of Monitoring Disease Progression Using Circulating Tumor DNA in Lymphoma: An Example From Primary Cutaneous DLBCL Leg-type

**DOI:** 10.1097/HS9.0000000000000690

**Published:** 2022-03-01

**Authors:** Christopher S. Trethewey, Harriet S. Walter, Abdullah N. M. Alqahtani, Ralf Schmid, David S. Guttery, Yvette Griffin, Matthew J. Ahearne, Gerald S. Saldanha, Sandrine P. N. Jayne, Martin J. S. Dyer

**Affiliations:** 1 Ernest and Helen Scott Haematological Research Institute, Leicester Cancer Research Centre, University of Leicester, United Kingdom; 2 Leicester Cancer Research Centre, University of Leicester, United Kingdom; 3 University Hospitals of Leicester NHS Trust, Leicester, United Kingdom; 4 Department of Molecular and Cell Biology, University of Leicester, United Kingdom; 5 Leicester Institute of Structural and Chemical Biology, University of Leicester, United Kingdom

Chemotherapy-refractory diffuse large B-cell lymphoma (DLBCL) remains a significant clinical problem. The ability to predict patients likely to have poor outcomes with conventional therapies may facilitate rational use of alternative, targeted treatment approaches before fulminant relapse. The utility of circulating tumor DNA (ctDNA) in DLBCL is currently being investigated. Initial studies have reported high levels of sensitivity for detection of residual disease, outperforming imaging techniques such as ^18^FDG-PET/CT scans.^1^ For example, Alizadeh et al recently demonstrated the feasibility of CAncer Personalized Profiling by deep Sequencing (CAPP-Seq) approach in DLBCL; the mean elapsed time between the first ctDNA-positive time point and radiological relapse was 188 days.^2^ Kurtz et al detected pretreatment ctDNA in 98% of patients with DLBCL receiving treatment with frontline or salvage immunochemotherapy.^3^ Early and major molecular responses after 1 and 2 cycles of treatment, resulted in superior outcome at 24 months. However, only 9% and 23% of patients within these validation sets had stage I and II disease, respectively. Limitations to the use of ctDNA to detect early stage malignancy have been identified; for example, in lung, where low-volume disease cannot be reliably detected using mutation profiles in ctDNA.^4^ Furthermore, some specific subtypes of disease (lung adenocarcinoma) do not shed detectable ctDNA into the peripheral blood.^5^

Primary cutaneous diffuse large B-cell lymphoma - leg type (PCDLBCL-LT) is a rare but distinct form of aggressive B-cell lymphoma, which is often resistant to therapy and associated with a poor prognosis.^6^ We have studied a case of chemotherapy and radiotherapy-refractory PCDLBCL-LT that remained localized to the lower leg for 13 years before systemic dissemination. We evaluated sequential plasma-derived ctDNA samples over 3 years during 3 different treatment modalities but were only able to detect ctDNA transiently, when the patient was taking the Bruton’s tyrosine kinase inhibitor, ibrutinib, and not during 2 episodes of systemic relapse.

A previously well 78-year-old gentleman was diagnosed with PCDLBCL-LT in 2006 involving the left lower leg. He received 4 cycles of systemic immunochemotherapy (R-CHOP) followed by involved-field radiotherapy (40 Gy, 15 fractions) with rapid and complete response. The patient subsequently relapsed in 2010 with left leg cutaneous involvement only, adjacent to the previously treated sites, and received additional treatment with 4 cycles of R-CHOP and radiotherapy (40 Gy in 15 fractions), again attaining clinical response. Two further relapses in 2011 and 2012, again in the left leg were treated with radiotherapy alone (4 Gy, single fraction). The patient remained disease free for 4 years before experiencing a further localized cutaneous relapse, refractory to rituximab, gemcitabine, cyclophosphamide, vincristine, and prednisolone (R-GVCP). He commenced venetoclax in January 2017. A rapid and complete metabolic response was obtained,^7^ before he relapsed in January 2019 (Figure 1). The patient then received 3 weeks of ibrutinib but experienced further rapid clinical progression. In April 2019, he commenced treatment on a phase 1 trial with a bispecific antibody, obtaining a complete metabolic response after 2 cycles; however, he progressed once again in January 2020.

**Figure 1.**
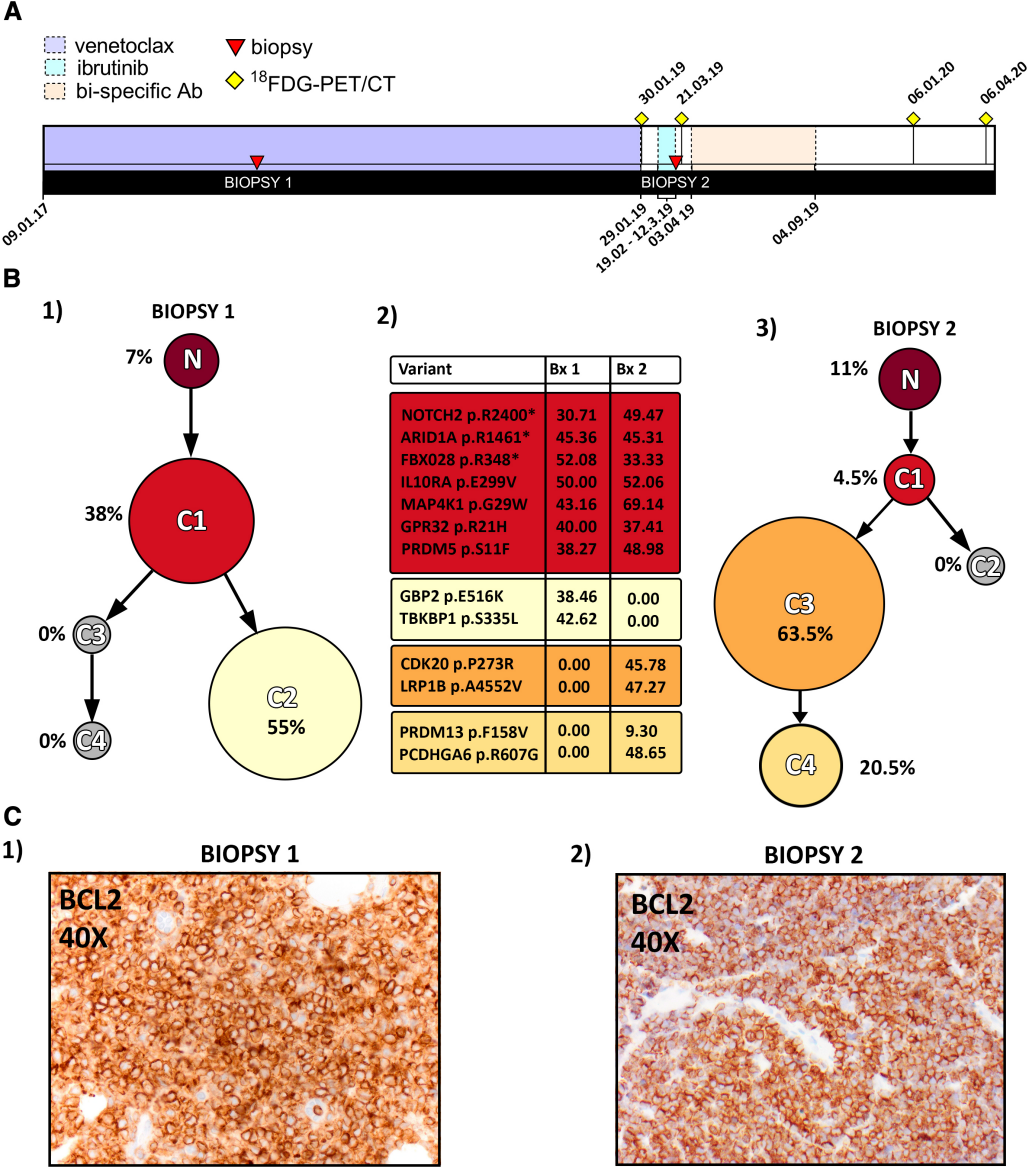
**Mutational and phylogenetic analysis of tumor biopsies at points of relapse on venetoclax and Ibrutinib.** (A) Timeline of clinical course. The patient received single agent venetoclax at a dose of 400 and 800 mg OD on alternating days from January 2017 until January 2019 (violet timeline). An excisional biopsy was during treatment with venetoclax (biopsy 1); biopsy 1 mutational data are shown in Suppl. Table S1. Following relapse, he was treated with single agent ibrutinib at a dose of 480 mg bd for a period of 3 weeks (pale blue timeline) but progressed rapidly with enlarging left inguinal lymph nodes (biopsy 2, WES). He then received treatment with a T cell–engaging bispecific antibody (orange timeline) and once again entered a ^18^FDG-PET/CT scan negative complete remission only to relapse 8 months after completion of therapy. (B) Clonal evolution in PCDLBCL-LT; clonal frequencies inferred from WES. Figure 2B1—analysis of biopsy 1 showed 2 clonal populations C1 (38%) and C2 (55%) with a small population of residual normal cells (7%). Figure 2B3 in contrast showing analysis of biopsy 2 with no detectable C2 cells and only 4.5% of C1. Two new subclones C3 and C4 comprising 63.5% and 20.5% of all cells were detected. Figure 2B2 shows possible driver mutations in the inferred subclones and percentages of cells carrying the different mutations, with further expansion of clonal mutations observed in Suppl. Figure S3. (C) Immunohistochemistry staining showing maintained BCL2 expression in biopsy 2 following 2 years venetoclax therapy. WES = whole exome sequencing.

Tumor DNA from freshly excised biopsy tissue and constitutional DNA from buffy coat were extracted using the DNeasy Blood and Tissue kit (Qiagen). Cell-free DNA (cfDNA) was isolated from blood plasma using the QIAamp Circulating Nucleic Acid Kit (Qiagen). Samples were quantified and quality tested using Qubit fluorometer (Thermofisher and Agilent Tapestation (Santa Clara, California, United States), respectively. Whole exome sequencing (WES) of tumors was carried out using hybridization capture-based Agilent Sure Select all exon target enrichment on Illumina to Novoseq 6000 by Novogene, to a minimum of 100× after deduplication. Targeted deep sequencing of plasma was performed following the Roche SeqCap-EZ workflow with KAPA HyperPrep library generation, captured with a custom 80 gene panel, sequenced on an Illumina Novaseq to a minimum of 1000× deduplicated coverage. Variant detection in tumor DNA and plasma was performed using the genome analysis toolkit v4 Mutect2 caller against germline DNA derived from buffy coat.^8^ Clonal phylogeny between tumor samples were analyzed in “R”^9^ with package “CLOE.”^10^ Serial monitoring of plasma by *ARID1A c.4381C>T fs* variant and *NOTCH2 c.7198C>T fs* variant mutant TaqMan assays was performed using the BIORAD Q×200 digital droplet polymerase chain reaction (PCR) system (BIORAD).^11^

WES of excision biopsies sampled at each episode of progression throughout the study was performed (Figure 1A). There were no activating mutations in the B-cell receptor (BCR) signaling pathway (*MYD88*, *CD79A/B*, or *CARD11*), as commonly reported in PCDLBCL-LT.^12–14^ However, a previously unreported G29W mutation within the ATP binding site of the *MAP4KI/HPK1* kinase was identified. From modeling using crystal structures, this mutation would impair MAP4K1 activity by reducing ATP binding. MAP4K1 has been shown to be a critical regulator of BCR signaling.^15^ Other truncal, potential genetic driver aberrations included *ARID1A* (COSM4031017), *NOTCH2* (COSM36210), and *FBXO28* missense mutations, as well as biallelic deletion of *RB1* and *CDKN2A*/B with monoallelic loss of *TP53* with the remaining *TP53* allele retaining germline sequences. Clonal phylogeny across biopsy samples prevenetoclax and following progression on ibrutinib treatment is summarized in Figure 1B.

Interestingly, 2 subclones were detected by WES before starting venetoclax, C1 and C2 (Figure 1B.1). Subclone C1 evolved resulting in the derivation of 2 new subclones C3 and C4, representing the acquisition of 34 previously undetected mutations (Figure 1B.2, B3). No *BCL2* mutations nor new mutations involved in regulation of apoptosis were detected, and levels of BCL2 expression were maintained on immunohistochemistry despite treatment with venetoclax for 2 years (Figure 1C). Following relapse on venetoclax,^7^ to enhance detection of residual disease, we analyzed sequential ctDNA samples. Serial plasma samples were analyzed by ddPCR over a 3-year period for the ARID1A p.R1461* and NOTCH2 p.R2400* nonsense variants, present in all biopsy samples, which are likely truncal mutations (Figure 2); 1 plasma sample was additionally assessed by CAPP-Seq during treatment on venetoclax (Figure 2B). Before and after starting venetoclax, no ctDNA harboring the *ARID1A* nor *NOTCH2* mutations could be detected by ddPCR or CAPP-Seq. This remained the case after the disease had spread to the left inguinal nodal region before starting ibrutinib (420 mg OD, Figure 2A.1). Surprisingly, higher levels of ctDNA (600 HHGE/ml plasma) were readily detectable 1 week later (Figure 2B). There were no detectable B cells in the peripheral blood by flow cytometry before starting ibrutinib and no detectable egress of tumor cells into the peripheral blood as assessed by sequential full blood counts (data not shown). Ibrutinib therapy was discontinued after 3 weeks due to rapid clinical progression. After 4 weeks washout and reassessment, the patient was started on experimental therapy (Figures 1A and 2B). By this time, levels of the *ARID1A* and *NOTCH2* mutations in ctDNA had fallen considerably in the face of disease progression, and again became undetectable following treatment on trial, remaining undetectable at subsequent relapse in January 2020 (data not shown).

**Figure 2.**
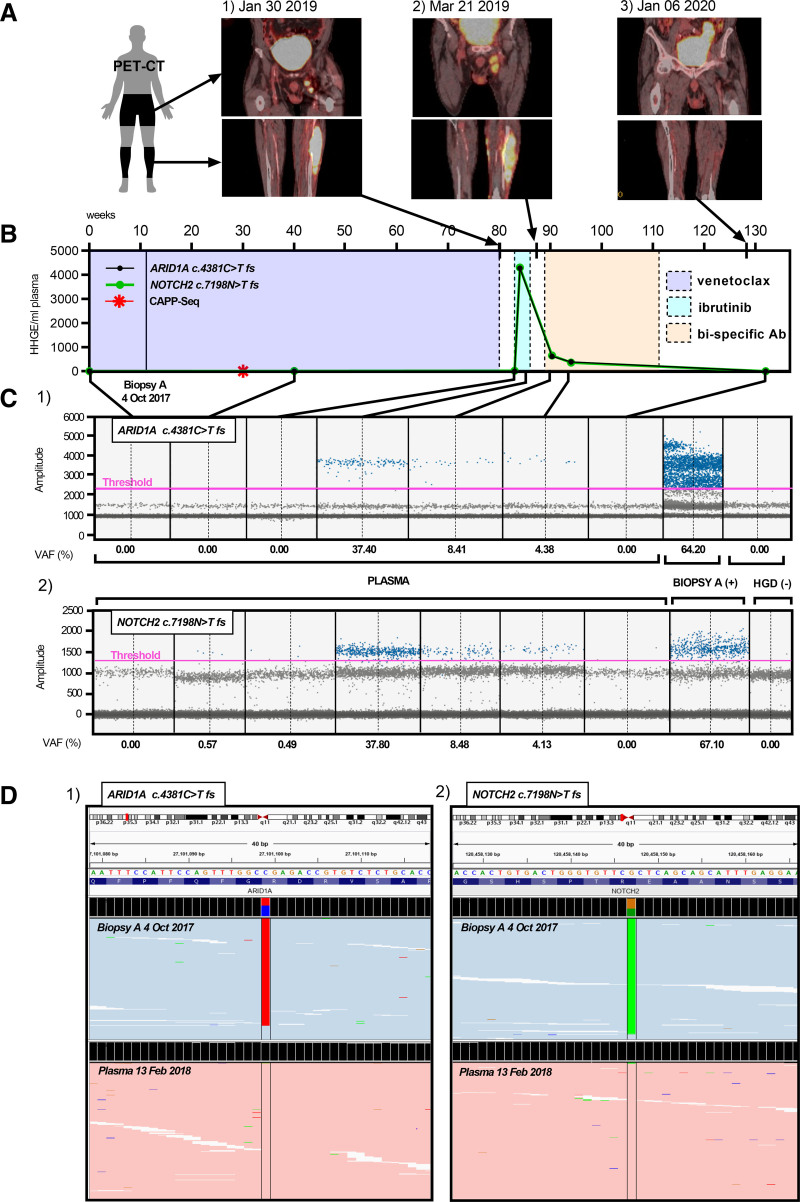
**Serial analysis of ctDNA by ddPCR and CAPP-Seq through treatment with Venetoclax, Ibrutinib and bispecific antibody.** (A) ^18^FDG-PET/CT scans taken at relapse following (1) venetoclax, (2) ibrutinib, and (3) bispecific /PD-L1 antibody therapy showing lumbar and lower leg regions. (B) Timeline in weeks of 7 plasma samples, analyzed for *ARID1A c.4381C>T* variant and *NOTCH2 c.7198C>T* variant by ddPCR assay throughout treatment, quantified as human haploid genomic equivalents per mL of plasma (HHGE/mL). Arrows showing points of ^18^FGD-PET/CT scans, dotted-lines marking treatment intervals, solid line indicating timepoint of biopsy A, and red star indicating additional plasma sample analyzed by CAPP-Seq during treatment on venetoclax. (C) Droplet frequency plots corresponding to (B) detected with *ARID1A* assay (C1, Black) and *NOTCH2* assay (C2, Green) of 7 serial plasmas, with additional biopsy A positive and HGD negative controls. Pink line indicating amplitude thresholds. Mutant droplets indicated in blue; negative droplets gray. (D) IGV plot of processed bam files for CAPP-Seq of biopsy A (blue) and plasma (pink) indicated in this figure, showing stacked and sorted reads for the *ARID1A c.4381C>T* variant (D1, red stacked reads) and *NOTCH2 c.7198C>T* variant (D2, green stacked reads). Plasma samples obtained 4 months after biopsy A showing *ARID1A c.4381* site and *NOTCH2 c.7198* site with no mutations and biopsy A harboring the *ARID1A c.4381C>T fs* variant and *NOTCH2 c.7198C>T fs* variant. HGD = human genomic DNA; IGV = Integrated genome viewer.

Monitoring of ctDNA levels in DLBCL may have a transformational role in predicting response and relapse. However, whether this is universally the case, across all biological subtypes and stages of DLBCL is not clear. From the example of solid tumors, specific subtypes of disease may not be associated with detectable levels of ctDNA and datasets studied in DLBCL include only small numbers presenting with early stage disease. In this case of PCDLBCL-LT, no ctDNA was detectable throughout the patients’ clinical course including following 2 systemic relapses. For reasons that are unclear, ctDNA was only detectable, while the patient was taking ibrutinib; whether this is a reproducible feature in other patients with DLBCL receiving Bruton’s tyrosine kinase inhibitor is not known. Other methods of disease monitoring may be necessary for some DLBCL subtypes and relying on a singular mutational approach to monitor low level disease may be ineffective.

## ACKNOWLEDGMENTS

We thank our patient for his kind perseverance and the staff of the Hope Clinical Trials Facility for their kind help. We thank Dr Paresh Sewpaul (Janssen Pharmaceuticals) for his kind help in obtaining compassionate use ibrutinib for this case. This study was approved by the University Hospitals of Leicester NHS Trust Research and Development (UK; 06/Q2501/122). Written informed consent from the patient was obtained for publication purposes.

## AUTHOR CONTRIBUTIONS

CST designed research, performed research, analyzed data, and wrote the article. RS and ANMA contributed data. HSW, DSG, YG, MJA, GSS, SPNJ, and MJSD wrote the article and supervised the study.

## DISCLOSURES

The authors have no conflicts of interest to disclose

## SOURCES OF FUNDING

This work was supported by Cancer Research UK in conjunction with the UK Department of Health on an Experimental Cancer Medicine Centre grant [C10604/A25151] and grants from Hope Against Cancer, CRUK, Leicester Haematology Research Fund and the Scott-Waudby Charitable Trust.

## Supplementary Material


