# Supplementary figures and images for: Limitations of Monitoring Disease Progression Using Circulating Tumor DNA in Lymphoma: An Example From Primary Cutaneous DLBCL Leg-type
Source: Hemasphere. 2022 Mar 1;6(3):e690. doi: 10.1097/HS9.0000000000000690 (PMC8893288; doi:10.1097/HS9.0000000000000690)

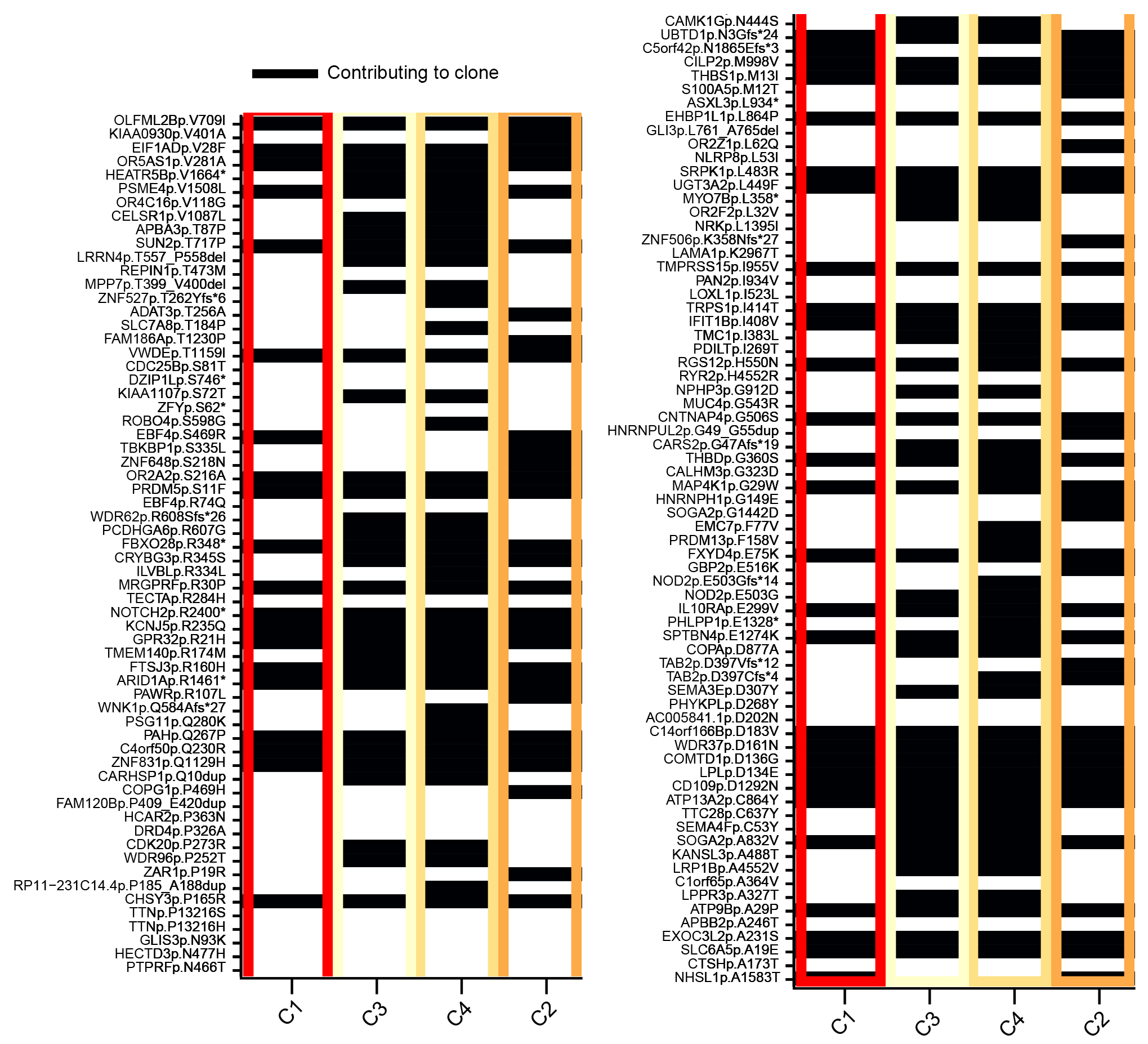

Supplement: Supplementary file 2 [file hs9-6-e690-s002.tif]
